# Microbiological Hazards, Not Undeclared Allergens, Dominate United States Food Recalls: An Analysis of 24,761 Product Recall Records, 2013–2025

**DOI:** 10.3390/foods15173115

**Published:** 2026-09-02

**Authors:** Wei Zhang, Catherine W. Y. Wong, Brian Schaneberg

**Affiliations:** Institute for Food Safety and Health, Illinois Institute of Technology, Bedford Park, IL 60501, USA; cwong21@illinoistech.edu

**Keywords:** food safety, food recalls, foodborne illness, foodborne pathogens, undeclared allergens, food surveillance, risk assessment, regulatory surveillance

## Abstract

Two claims dominate popular discussion of US food safety: (1) Food recalls are rising year over year, and (2) undeclared allergens have become the leading cause. In this study, we aimed to (1) test both claims against the openFDA Food Enforcement bulk export for fiscal years (FY) 2013–2025, the post-FSMA-core period following implementation of the FDA Food Safety Modernization Act (FSMA), including 24,761 conventional human-food product recall records; (2) identify the dominant microbiological hazards and undeclared allergens that drive FDA recalls; (3) compare the FDA recall record with USDA Food Safety and Inspection Service (FSIS) recalls of meat, poultry, and egg products; and (4) evaluate potential correlations between FDA and FSIS recall activity and the foodborne-illness burden recorded in CDC’s National Outbreak Reporting System (NORS). Product recall records were recoded into five hazard categories using a validated 70-rule keyword scheme. Our analysis showed that annual product recall records did not increase (mean falling from 2403/yr in FY2013–2017 to 1593/yr in FY2018–2025, Welch t = 3.08, *p* = 0.024), and microbiological hazards were the largest category in 12 of 13 years, exceeding allergens 1.7-fold cumulatively. *Listeria monocytogenes* was a major driver of microbiological food recalls concentrated in fresh produce, ice cream/frozen desserts, and dairy/cheese; whereas undeclared milk was by a wide margin the most-cited allergen in food recalls concentrated in dairy/cheese, ice cream, and bakery products. Compared to FDA recalls, FSIS recalls were fewer, more severe (68.6% vs. 45.6% Class I), and allergen-led, reflecting differing inspection frameworks rather than a uniform allergen surge. Overall, food recalls and foodborne outbreak illnesses did not display a significant correlation (Spearman rho = 0.30). Classification lag shortened over the period (rho = −0.32, *p* < 0.001), a process-efficiency gain that can masquerade as rising recall activity in count-based trackers. Across all four objectives, the recall record describes a maturing, severity-weighted surveillance system rather than a deteriorating food supply.

## 1. Introduction

Foodborne disease remains a substantial and largely preventable public-health burden in the United States, where contaminated food is estimated to cause roughly 48 million illnesses, 128,000 hospitalizations, and 3000 deaths each year [1,2], with an associated economic cost in the tens of billions of dollars annually [3]. Within this landscape, the product recall is the principal mechanism by which unsafe food is removed from commerce once a hazard is identified. Because recalls are logged, classified by severity, and released as open data, they also serve as one of the most visible and continuously updated signals of food safety performance available to regulators, industry, and the public. How that signal is read, whether recalls are taken as evidence of a deteriorating food supply or of a maturing surveillance system, shapes consumer confidence, regulatory priorities, and legislative attention.

The modern US food-safety framework rests on a series of statutory reforms. The FDA Food Safety Modernization Act (FSMA) of 2011 [4] reoriented federal oversight from reacting to contamination toward preventing it, with its foundational preventive-controls and produce-safety rules phasing into force over the following years; fiscal year (FY) 2013 onward therefore represents a ‘post-FSMA-core’ regime in which reporting and classification practices were comparatively stable. In parallel, allergen oversight expanded through the Food Allergen Labeling and Consumer Protection Act (FALCPA) of 2004 [5] and the Food Allergy Safety, Treatment, Education, and Research (FASTER) Act of 2021 [6], which codified and then broadened the set of major allergens subject to mandatory labeling. Regulatory jurisdiction is split: FDA oversees most of the food supply, while meat, poultry, and processed-egg products fall to the USDA Food Safety and Inspection Service (FSIS) [7], and FDA’s own food programs were consolidated into a unified Human Foods Program in 2024 [8]. These overlapping reforms make the recall record an informative but complicated object of study, because changes in counts can reflect changes in rules and reporting as readily as changes in underlying risk.

A central aim of FSMA was preventive: requiring facilities to anticipate and control hazards before product reaches commerce [9]. FSMA’s success would be measured not only by fewer foodborne illnesses but, plausibly, by fewer and less severe recalls over time. The cornerstone rule, Preventive Controls for Human Food, requires covered facilities to implement a written food-safety plan developed by a Preventive Controls Qualified Individual (PCQI) to identify known or reasonably foreseeable biological, chemical (including allergen), and physical hazards [4]. Recalls nonetheless continue to occur across all of these hazard classes, which motivates the empirical questions addressed here: across the post-FSMA-core period, have recall frequency and severity declined, and how have the major hazard categories (refined in this analysis into microbiological, allergen, chemical, physical, and other-quality groupings) trended over time?

Against this backdrop, two claims recur in trade press, advocacy, and public commentary. The first is that US food recalls are rising year over year: recent reporting has emphasized that recall counts have climbed to multi-year highs and that the volume of recalled product has surged sharply between quarters [10,11,12,13]. The second is that undeclared allergens have displaced microbiological contamination as the leading cause of recalls; widely circulated analyses have described undeclared allergens as the single most common reason for recall, in some years accounting for roughly half of all events [14,15,16]. Both claims are intuitively plausible. FSMA and expanded labeling enforcement increased the visibility of allergen-related violations [17,18,19], and individual years have indeed seen allergen recalls spike, driven in part by the 2023 addition of sesame as a major allergen [14], while high-profile microbiological events such as the 2024 *Listeria* outbreak linked to sliced deli meats [20] keep pathogens in the headlines without necessarily dominating the counts. Both claims also carry policy weight. They are invoked to justify expanded labeling enforcement, to characterize the food supply as increasingly hazardous, and to set surveillance priorities [21]. Yet both rest largely on visual inspection of raw counts rather than formal statistical testing, and counting artifacts (shifts in reporting completeness, classification lag, or a single anomalous year) can easily masquerade as genuine trends [22,23].

Testing either claim first requires being explicit about what is being counted, because the recall record supports more than one unit of analysis and the two are easily conflated. FDA exposes the choice directly in its Data Dashboard, which allows recalls to be filtered at either the event or the product level. An event is a firm’s recall of one or more products, so an event-level count reports how many recalls occurred, whereas a product-level count reports how many individual products were recalled [24]. In the openFDA Food Enforcement export these two levels correspond to two identifier fields. A recall event is a single firm’s recall action, grouped by event_id. A product recall record is one individual product or package size listed under that action, carrying its own recall_number; because the export delivers one row per recall_number, the product recall record is also what a naive count of the file returns. Notably, the openFDA field reference describes both identifiers with identical wording—each is given as a numerical designation assigned by FDA to a specific recall event for tracking purposes—so the documentation itself does not distinguish them [25]. A third quantity, the physical amount recalled, is held as free text in product_quantity and is not a count at all. Since one event may span dozens of product recall records, the event and product counts differ by roughly 3.7-fold in the present dataset, and a trend, share, or ratio computed on one unit is not interchangeable with the same statistic computed on the other. Unless recall events are named explicitly, all counts reported in this paper are product recall records.

Prior analyses of the bulk recall record have been valuable but largely descriptive. DeBeer et al. characterized FDA-regulated food recalls from 2002 to 2023 [26], and a companion study compared USDA and FDA recalls over a similar span [27]; both summarized counts and categories but stopped short of testing the specific ‘increasing’ and ‘allergen-dominant’ propositions with trend statistics, and neither isolated the post-FSMA-core window or triangulated recall counts against independent outbreak-outcome data. The openFDA Food Enforcement export [25,28,29] now makes such testing feasible at scale, and pairing it with the US Centers for Disease Control and Prevention (CDC) National Outbreak Reporting System [30] and the USDA-FSIS recall record [7] allows the recall signal to be checked against the illnesses, hospitalizations, and deaths it is meant to forestall.

We therefore set out to test, rather than assert, the dominant claims about US food recalls and to place them in a fuller analytical context, pursuing four objectives. First, we tested the two popular claims directly against the openFDA bulk record: whether total recalls increased across the post-FSMA-core window of FY2013–2025, and whether allergen hazards overtook microbiological hazards as the leading recall category. Second, we identified the specific microbiological hazards and undeclared allergens responsible for FDA recalls, characterizing the pathogens, allergens, and product categories that drive the headline patterns, with particular attention to *Listeria monocytogenes*, which has the highest case-fatality rate of any common foodborne pathogen and accounted for the largest share of outbreak-associated foodborne deaths in the surveillance window examined here, although non-typhoidal *Salmonella* and *Toxoplasma gondii* are estimated to cause more foodborne deaths nationally in absolute terms [2,31]. Third, we compared the FDA recall record with the USDA-FSIS record for meat, poultry, and egg products—two regulatory systems built on different inspection frameworks. Fourth, we tested whether FDA and FSIS recall activity correlates with the illnesses, hospitalizations, and deaths captured in CDC’s National Outbreak Reporting System. This was done to also determine whether recall counts can serve as a proxy for foodborne-disease burden. As detailed below, both popular claims fail under formal testing, the two recall systems differ in instructive ways, and recall activity proves to be a measure of regulatory response to severe, traceable hazards rather than of population illness.

## 2. Materials and Methods

### 2.1. Data Sources

Records were drawn from the openFDA Food Enforcement bulk export (https://download.open.fda.gov/food/enforcement/food-enforcement-0001-of-0001.json.zip) downloaded on 9 May 2026. The full export contains 28,784 records spanning 2004–2026; after removing five cosmetic recalls, two animal/pet-food recalls, and 1804 dietary-supplement recalls (the food enforcement endpoint carries human-food recalls; FDA publishes animal and veterinary product recalls separately, so only isolated animal-food entries appear in this export), a total of 26,973 conventional human-food records remained. The federal fiscal year (FY) is defined as 1 October of the prior calendar year through 30 September. Two units of analysis are used throughout this paper. A recall event is a single firm’s recall action, identified in the export by event_id; a product recall record is an individual product or package size listed under that action, identified by recall_number. A recall event may comprise one or more product recall records, and unless stated otherwise all counts reported here are product recall records. Recalls were anchored on recall_initiation_date (the date the firm took action), not report_date or center_classification_date [25,28]. Recall data of meat, poultry, and processed-egg products were retrieved from the USDA Food Safety and Inspection Service (FSIS) recall record [7]. FSIS records were obtained from an archived snapshot of the FSIS recall database (capturing recall title, date, number, classification, reason, affected states, and the press-release summary text), restricted to the same FY2013–FY2025 window, and recoded with the identical 70-rule hazard scheme applied to the FDA data. The FSIS snapshot available for download at the time of this analysis was dated 2 March 2025, which was the most recent archive obtainable; the remaining months of FY2025 were not available. FY2025 is therefore represented only partially in the FSIS series, and FSIS trend statistics should be read with that truncation in mind. Outcome data (illnesses, hospitalizations, and deaths) were taken from CDC NORS rather than from FSIS press-release text, which narrates counts inconsistently and cannot be aggregated reliably.

Foodborne-disease outcomes (illnesses, hospitalizations, and deaths) were drawn from the US Centers for Disease Control and Prevention (CDC) National Outbreak Reporting System (NORS) public-use foodborne-outbreak dataset [30], covering calendar years 2013–2023. NORS catalogues investigated outbreak clusters rather than individual recalls; it was therefore aligned to the recall data conceptually, at the pathogen-by-year and food-vehicle level, rather than by one-to-one record linkage. Per-pathogen and per-year aggregates, not per-event joins, are the credible unit of comparison, and reported hospitalization and death counts, contributed voluntarily by states, should be read as lower bounds.

### 2.2. Hazard Recoding

FDA’s native categorization is inconsistent: heavy metals and mycotoxins drift between buckets, and ‘foreign material’ is sometimes coded as physical and sometimes as microbiological depending on the writer. We recoded each recall into one primary hazard category using 70 ordered keyword rules applied to reason_for_recall (with product_description as fallback). The five categories were microbiological (pathogens, mold, infestation), allergen (undeclared Big-9 allergens, sulfites, mislabeling that creates allergen exposure, cross-contact), chemical (heavy metals, mycotoxins, pesticides, undeclared drugs/colors, natural toxins), physical (undeclared metal, plastic, glass, rubber, wood, bone), and other_quality (under-fill, packaging integrity, GMP violations without specific hazard, temperature abuse, mispackaging). Records that matched no rule were left as ‘uncategorized’ (*n* = 1899, 7.7% of cleaned records); the rule dictionary is preserved for audit.

### 2.3. Statistical Analyses

Trends in annual counts were tested with Mann-Kendall (tau, two-sided) and with a generalized linear count model of total recalls on year. Because annual recall counts are large (approximately 1000–2800 per year) while the series comprises only 13 annual observations, overdispersion of the Poisson specification was assessed formally using the Pearson chi-square/residual degrees-of-freedom ratio and the residual deviance/degrees-of-freedom ratio. The Poisson model proved severely overdispersed, so a negative binomial GLM (dispersion parameter estimated by profile maximum likelihood) was adopted as the primary count model, and a quasi-Poisson model with the scale parameter estimated from the Pearson statistic was fitted as a sensitivity check; relative fit was compared by AIC. Composition stability across years was tested with Pearson chi-square (hazard category × fiscal year). Whether allergen exceeded microbiological more often than chance was tested with a one-sided binomial test (k of 13 years, null *p* = 0.5). Pre/post FY2017 means were compared with Welch’s *t*-test. Trend in classification lag (days from recall_initiation_date to center_classification_date) was tested with Spearman rank correlation. All analyses were run in Python 3 (pandas, scipy, statsmodels, pymannkendall) [32,33].

### 2.4. Hazard-Recoding Validation

The 70-rule keyword classifier was validated three ways (an internal multi-match audit, a stratified 300-record manual review showing 95.7% overall precision (every category at or above 90%), and an adversarial robustness test) and was proven sound. The finding does not depend on the residual 7.7% uncategorized bucket: even reassigning every uncategorized record to allergen leaves microbiological hazards ahead (11,443 vs. 8616), and correcting the identified classification errors widens rather than narrows the microbiological-over-allergen margin.

## 3. Results

### 3.1. Dataset Characteristics

After scope filters (Section 2.1), the analysis window contained 24,761 FDA food product recall records from 4649 unique recalling firms. The earliest recall_initiation_date in the full export was 12 July 2012 and the latest was 9 May 2026; the FY2013–FY2025 analysis window itself closes on 30 September 2025. Across the 24,761 eligible product recall records, the median classification lag (firm action to FDA classification) was 38 days and the 90th percentile was 126 days. Dataset characteristics are summarized in Table 1.

Two features of the cleaned dataset are important for interpretation. First, the scope filters (removal of cosmetics, animal/pet food, and dietary supplements) are what make this a conventional human-food series and account for much of the divergence from prior bulk analyses that did not separate these streams [26]. Second, the 38-day median classification lag paired with a 90th-percentile lag of 126 days signals a right-skewed distribution in which a minority of complex cases take far longer to classify, a pattern shown in the recall-duration analysis.

### 3.2. Statistical Analyses Do Not Support the Two Public Claims About FDA Food Recalls

#### 3.2.1. Total Food Recalls Did Not Increase

**Figure 1:**
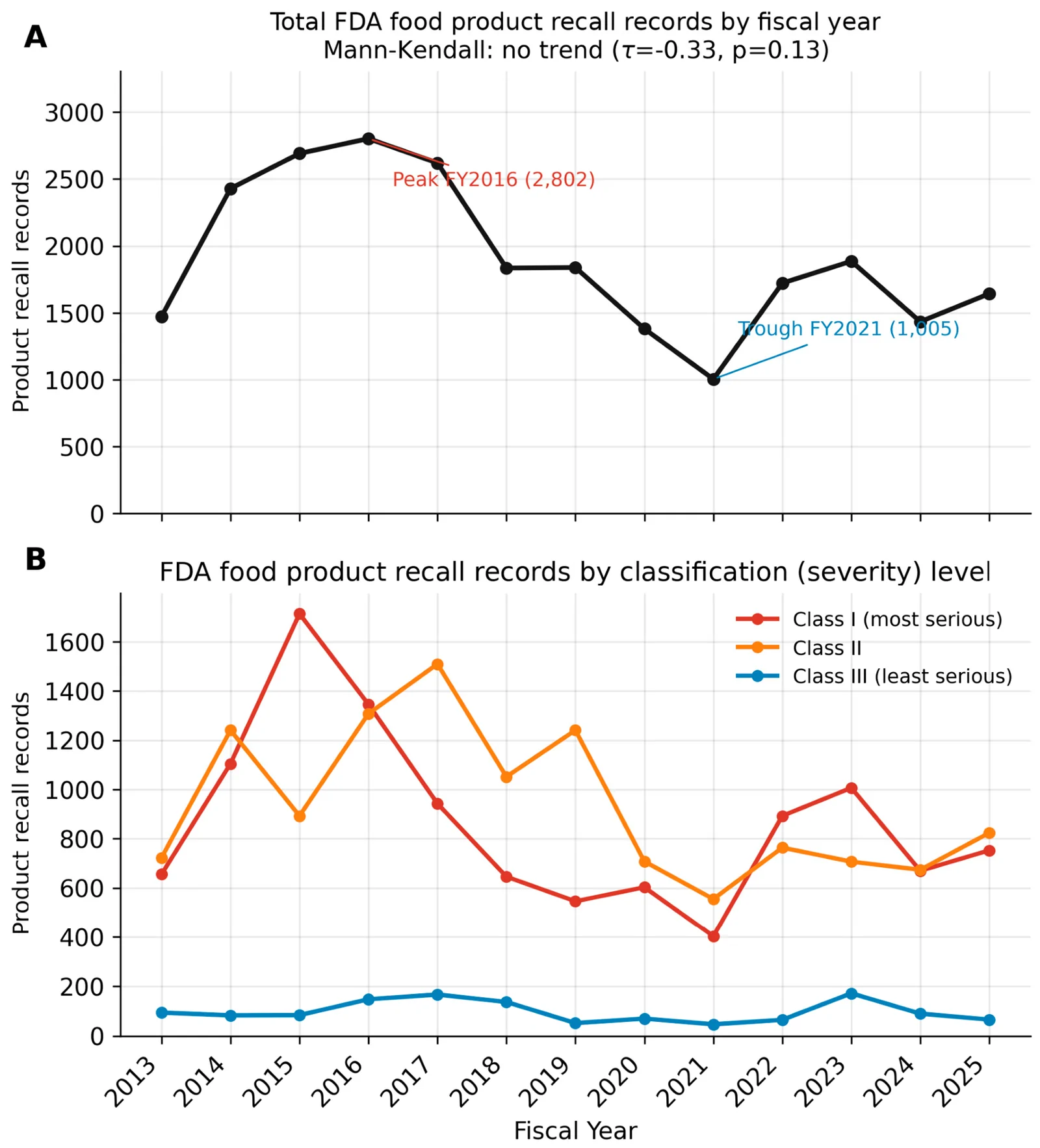
FDA food recall trends, FY2013–2025 (conventional human food; dietary supplements excluded). (**A**) Total product recall records per fiscal year; the series is non-monotonic, peaking in FY2016 and reaching a trough in FY2021, with no significant monotonic trend (Mann-Kendall). (**B**) Product recall records by FDA classification (severity) level; Class I and Class II carry most of the volume and track the overall trend, while Class III remains low throughout.

Total product recall records per fiscal year are shown in Figure 1A. The Mann-Kendall test on the 13-year series returned tau = −0.33 (z = −1.53, *p* = 0.13); the test did not reject a null of no monotonic trend, and the Sen’s slope estimator [34] (−85 records/year) is in fact negative. A Poisson GLM regressing total product recall records on year produced an incidence rate ratio (IRR) of 0.960 per year, but the model was severely overdispersed (Pearson chi-square = 1342.0 on 11 residual degrees of freedom; dispersion φ = 122.0; residual deviance/df = 129.3), so its nominal *p*-value (3.8 × 10^−126^) reflects the failure of the mean-equals-variance assumption at these count magnitudes and is not interpretable. Refitting the same trend as a negative binomial GLM, which fits the series far better (AIC 199.5 versus 1547.6; residual deviance/df = 1.19), returned IRR = 0.959 per year (95% CI 0.926–0.994, *p* = 0.020); a quasi-Poisson model with the scale estimated from the Pearson statistic returned IRR = 0.960 per year (95% CI 0.925–0.996, *p* = 0.031). All three specifications place the IRR below 1, indicating a modest year-on-year decline of roughly 4% rather than the increase asserted by the popular claim. The series peaked in FY2015 (2691) and FY2016 (2802) and reached a trough in FY2021 (1005). Mean product recall records fell from 2403/yr in FY2013–2017 to 1593/yr in FY2018–2025 (−33.7%; Welch t = 3.08, *p* = 0.024). Of the three classification levels, Class II showed the strongest downward tendency (tau = −0.35, *p* = 0.112, decreasing), but no severity tier reached statistical significance; Class I and Class III likewise showed no significant change.

Interpretation of the temporal pattern reinforces the formal trend test: the series rises through the FSMA compliance-phase years (FY2014–FY2016), then declines and plateaus below the pre-rule baseline rather than climbing monotonically as the popular ‘recalls are rising’ narrative would predict. The FY2021 trough (1005 records) coincides with pandemic-era disruption to inspection and firm operations, and the partial FY2022–FY2025 rebound returns the series only to roughly its FY2018–FY2019 level, not to the FY2015–FY2016 peak. Figure 1B shows this shape is carried mainly by Class I and Class II recalls while Class III stays low and flat, so the absence of a sustained post-FSMA increase holds across every severity tier.

The class-level counts clarify which severity tier drives the decline. Class I recalls are volatile but show no upward drift, peaking in FY2015 and again in FY2023; Class II recalls fall most clearly across the window, although this decline, like those in the other two tiers, does not reach statistical significance. The persistently small Class III counts indicate that the least-severe recalls are not inflating recent totals, so the flat-to-declining pattern cannot be dismissed as an artifact of minor recalls.

A recurring version of the ‘recalls are rising’ narrative holds that, even as the number of recall announcements has fallen in recent years, the amount of food being recalled has grown—fewer but bigger recalls. We tested this directly. The openFDA record allowed us to count three different terms that are easily confused: a recall event (one firm’s recall action), a recalled product (each individual product or package size listed under that action), and the recalled volume (how many cases, pounds, or units were involved). Measured consistently, the claim does not hold: recall events and recalled product recall records moved down together rather than apart, and the apparent growth in ‘volume’ is an artifact of how the data are recorded.

In the openFDA Food Enforcement file a single recall is identified at two levels: an event_id groups everything a firm recalls in one action, while each distinct product or package size receives its own recall_number. Over FY2013–2025 the 24,761 product recall records that anchor this study correspond to only 6674 distinct recall events—on average about 3.7 products per event. A third quantity, the physical amount recalled, is stored as free text in the product_quantity field (for example, ‘17,586 cases’ or ‘90/1 lb packages’). Treating these three as interchangeable is the root of the ‘fewer but bigger’ impression.

Both countable quantities trended down across the window, not in opposite directions. The number of recall events fell (Kendall tau = −0.385, *p* = 0.076; about 12 fewer events per year), as did the number of product recall records (tau = −0.333, *p* = 0.127; about 85 fewer per year). The two series are tightly coupled, rising and falling in step (Pearson r = 0.846, *p* < 0.001; Spearman rho = 0.775), and the number of products per event showed no meaningful trend (tau = −0.308). Comparing the first half of the period with the second (FY2013–FY2019 vs. FY2020–FY2025), mean annual events fell from 569 to 448 and mean annual product recall records from 2241 to 1512, while products per event was essentially unchanged (3.92 vs. 3.36). A claim that products rose while events fell requires these series to diverge; instead they are among the most strongly correlated pairs in the dataset (Table 2).

The volume field cannot support a reliable national trend, for four reasons. First, units are not comparable: parseable entries express the quantity in 246 distinct tokens—cases, units, pounds, bags, bottles, even ‘sandwiches’—that cannot be summed into a single total. Second, roughly 11% of records carry no usable quantity (blank or ‘Unknown for this product’). Third, and most important, the field frequently records the event-level total and then repeats it on every product line within the event: the largest single value in the file, TreeHouse Foods’ 9,907,389-case recall, appears on 65 separate product rows, so naively summing product_quantity counts it 65 times. This replication inflates the FY2025 case ‘total’ by roughly 42 times relative to a correct one-total-per-event count. Fourth, even after correcting for replication, volume is dominated by a handful of very large recalls, so the yearly total lurches with single events and shows no trend (corrected cases tau = 0.051, *p* = 0.858). Commercial recall ‘volume indexes’ that report surging units are measuring exactly these episodic mega-recalls, not a broad shift in the food supply.

Read consistently, the openFDA record does not support the ‘fewer but bigger recalls’ claim. Recall events and recalled product recall records declined together over FY2013–2025 and are strongly positively correlated; the number of products per event was flat; and recalled volume cannot be estimated reliably from openFDA and, where it can be approximated, shows no robust upward trend. The impression of rising volume amid falling counts arises from conflating three different units and from a small number of very large recalls whose event-level totals are replicated across many product recall records. This pattern is consistent with the study’s broader finding that recall activity tracks discrete, severity-driven events rather than a steadily growing hazard burden.

#### 3.2.2. Microbiological Hazards, Not Allergens, Dominate FDA Food Recalls

**Figure 2:**
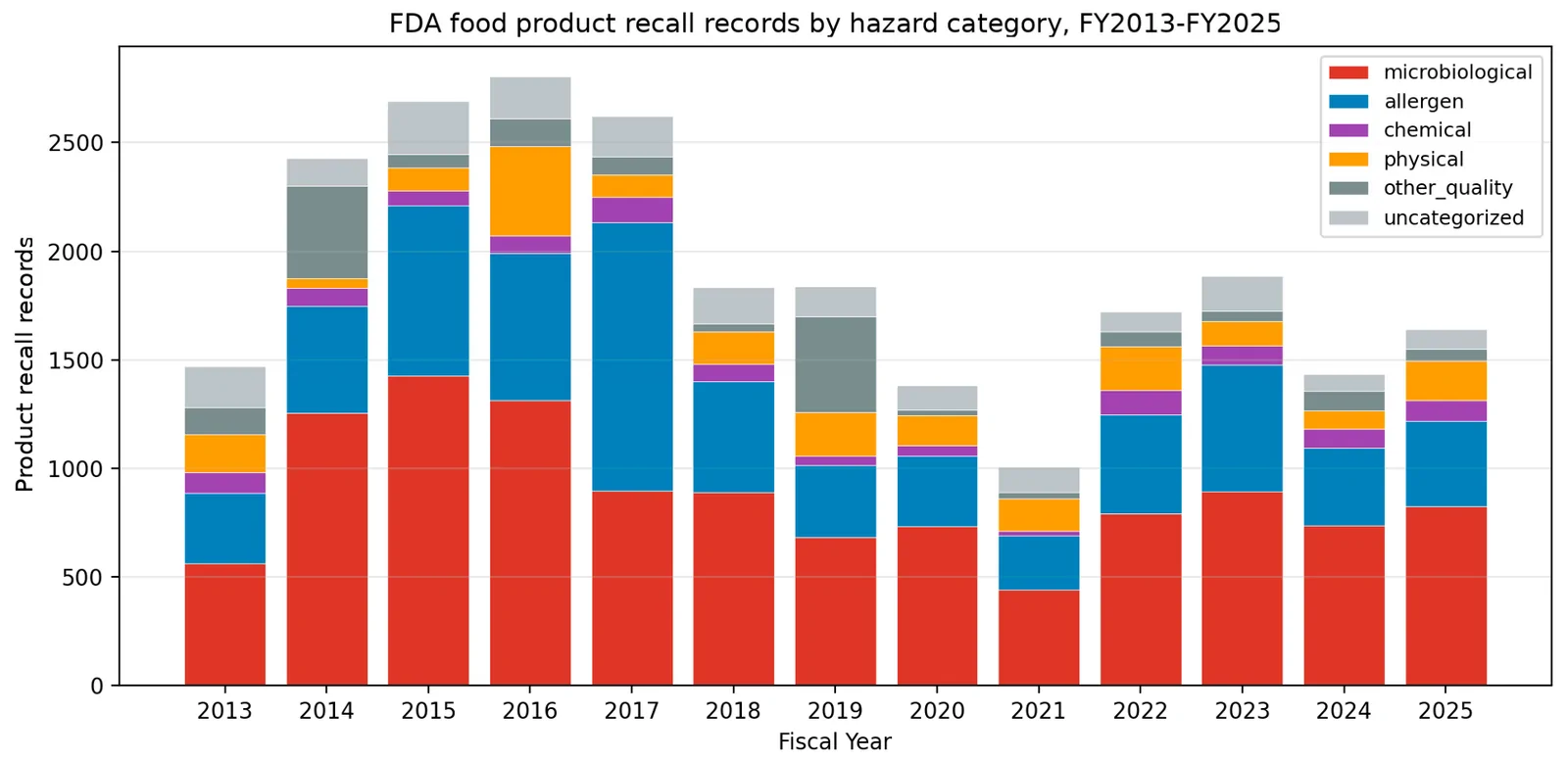
Annual product recall records by hazard category (stacked). Microbiological hazards (red) form the base layer in every year except FY2017.

**Figure 3:**
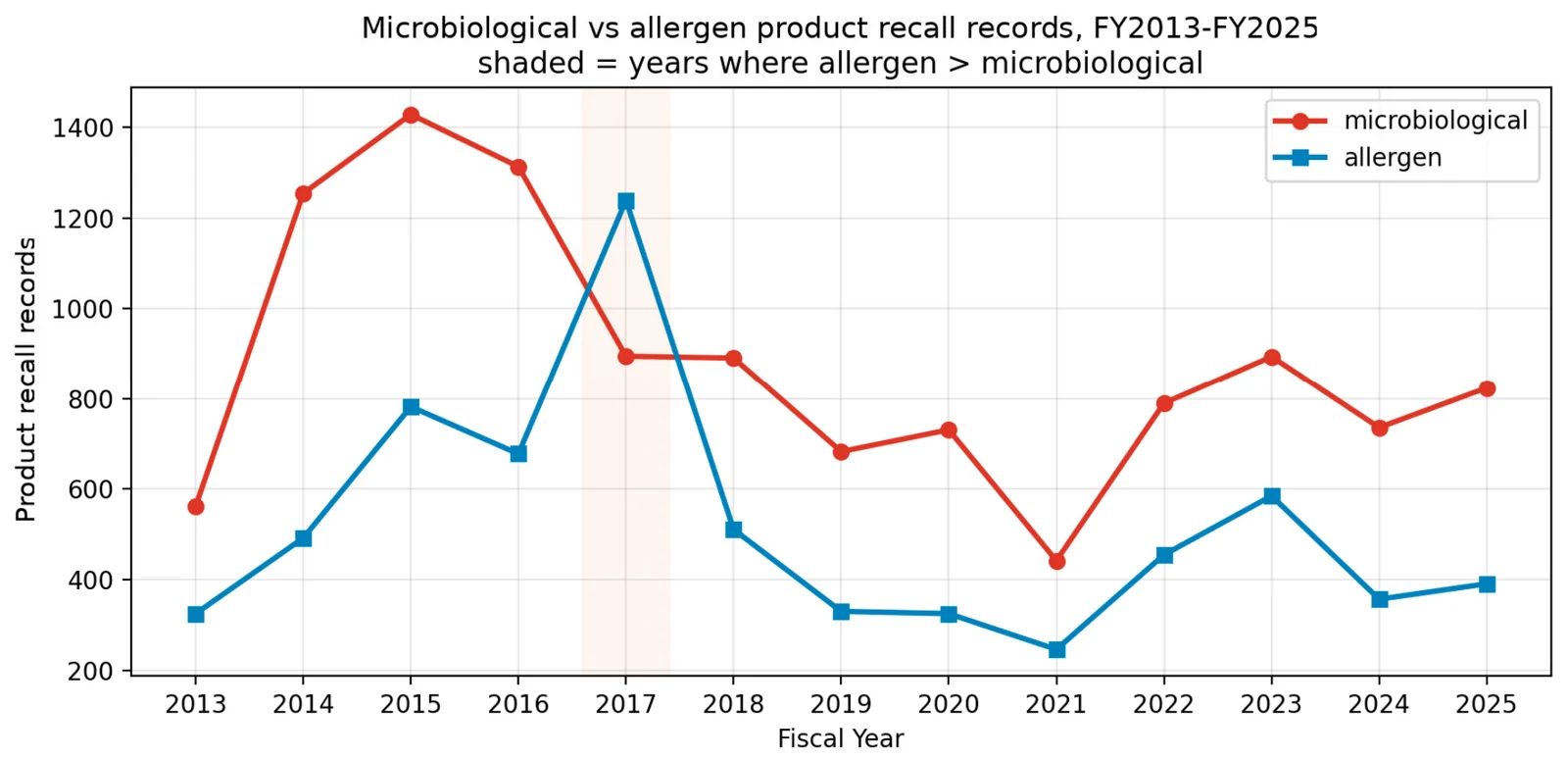
Direct comparison of microbiological and allergen recalls. Shading marks years where allergen exceeded microbiological—only FY2017.

The annual hazard composition is shown in Figure 2, and a direct comparison between microbiological hazards and allergens is shown in Figure 3. Microbiological hazards accounted for 11,443 product recall records (46.2%)—more than the allergen (6717, 27.1%) and chemical (1023, 4.1%) categories combined. Microbiological was the largest category in 12 of 13 fiscal years. Allergen exceeded microbiological in only 1 year (FY2017: 1239 vs. 895); a one-sided binomial test of allergen-dominance against a null of *p* = 0.5 yields *p* = 1, providing no support for the claim that allergens are the dominant cause. Hazard composition did vary by year (chi-square = 3084, df = 60, *p* < 0.001), but the variation does not imply rank inversion—microbiological dominance is robust across the window.

**Figure 4:**
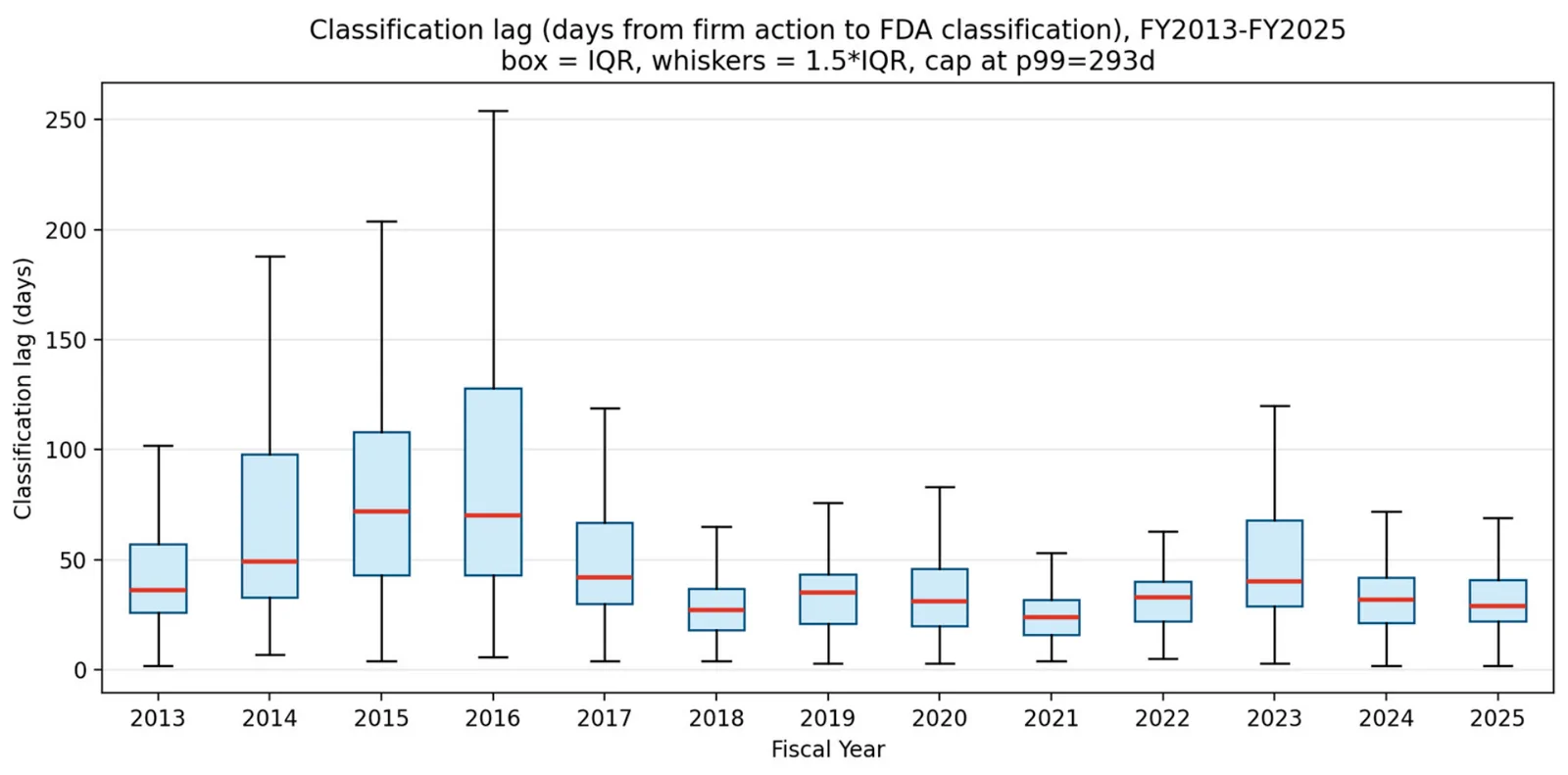
Classification lag by fiscal year (capped at p99 for visualization). Median lag fell from approximately 60 days in FY2013–2014 to under 30 days by FY2022–2024.

Classification lag, the calendar interval between recall initiation date and center classification date, declined over the analysis window (Spearman rho = −0.32, *p* < 0.001, n = 24,760). The narrowing distribution (Figure 4) is consistent with FDA process improvements rather than with rising recall pressure. This finding is policy-relevant: a rising count of ‘reported’ recalls in informal trackers can reflect faster FDA classification rather than more underlying safety events.

The lag distribution narrows most sharply between the early window (FY2013–FY2014) and FY2022 onward, and the compression of the upper whisker shows that even the slowest-to-classify recalls are resolved faster than a decade earlier. This is consequential for any tracker that counts recalls by report or classification date rather than by initiation date: as the agency clears its queue more quickly, a fixed underlying rate of firm actions appears as a rising count of newly classified recalls, manufacturing an illusion of increasing recall activity.

Modeling the classification lag directly confirms the descriptive trend reported above. The median lag was 38 days (interquartile range 26–63). In a quantile regression holding fiscal year, final classification, and voluntary/mandated status constant, the largest effect was FDA-mandated status, mandated recalls were classified 43 days later than voluntary ones, while microbiological recalls took modestly longer than allergen recalls (+3.9 days, plausibly awaiting laboratory confirmation), and each successive fiscal year shaved about 1.9 days off the median lag, an outlier-robust acceleration corroborated by a negative-binomial mean model (incidence-rate ratio 0.942 per year). The implication is the same as the descriptive finding: a rising count of newly classified recalls can partly reflect FDA classifying faster rather than more underlying safety events.

### 3.3. Major Microbiological Hazards and Undeclared Allergens Driving FDA Recalls

**Figure 5:**
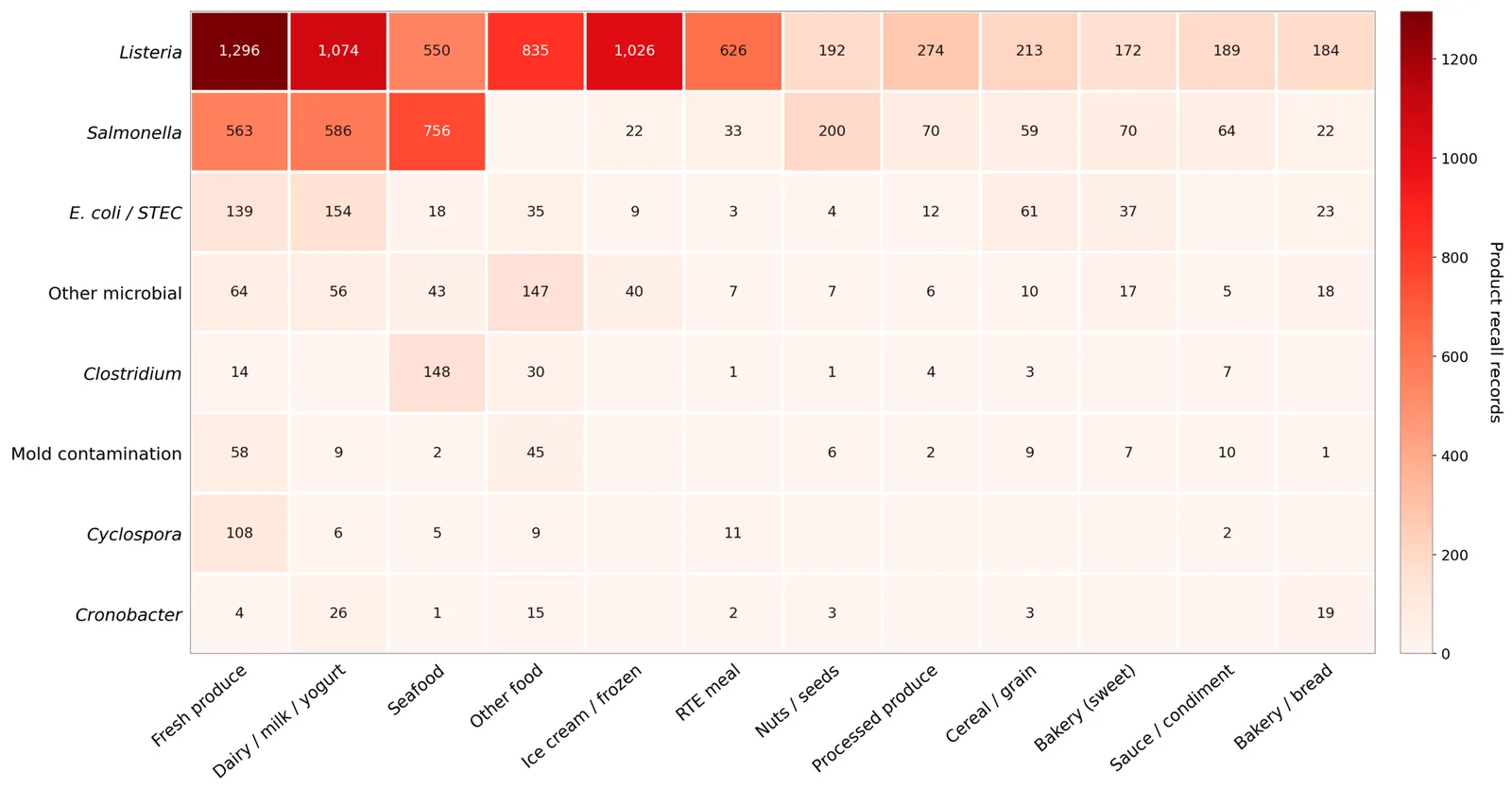
Microbiological recalls cross-tabulated by specific pathogen and product category, FY2013–2025.

The pathogen-by-product structure in Figure 5 explains why microbiological hazards dominate the record. *L. monocytogenes* alone accounts for 6858 of the 11,443 microbiological product recall records across FY2013–FY2025 and concentrates in fresh produce, ice cream/frozen desserts, and dairy/cheese—refrigerated, ready-to-eat categories where post-process contamination and cold-chain persistence are the defining risks [31]. *Salmonella* forms a second, distinct cluster in seafood, dairy, and produce. With dietary supplements removed from scope, *Cronobacter* no longer concentrates in a single category, indicating that earlier supplement-heavy analyses likely overstated its infant-formula concentration.

**Figure 6:**
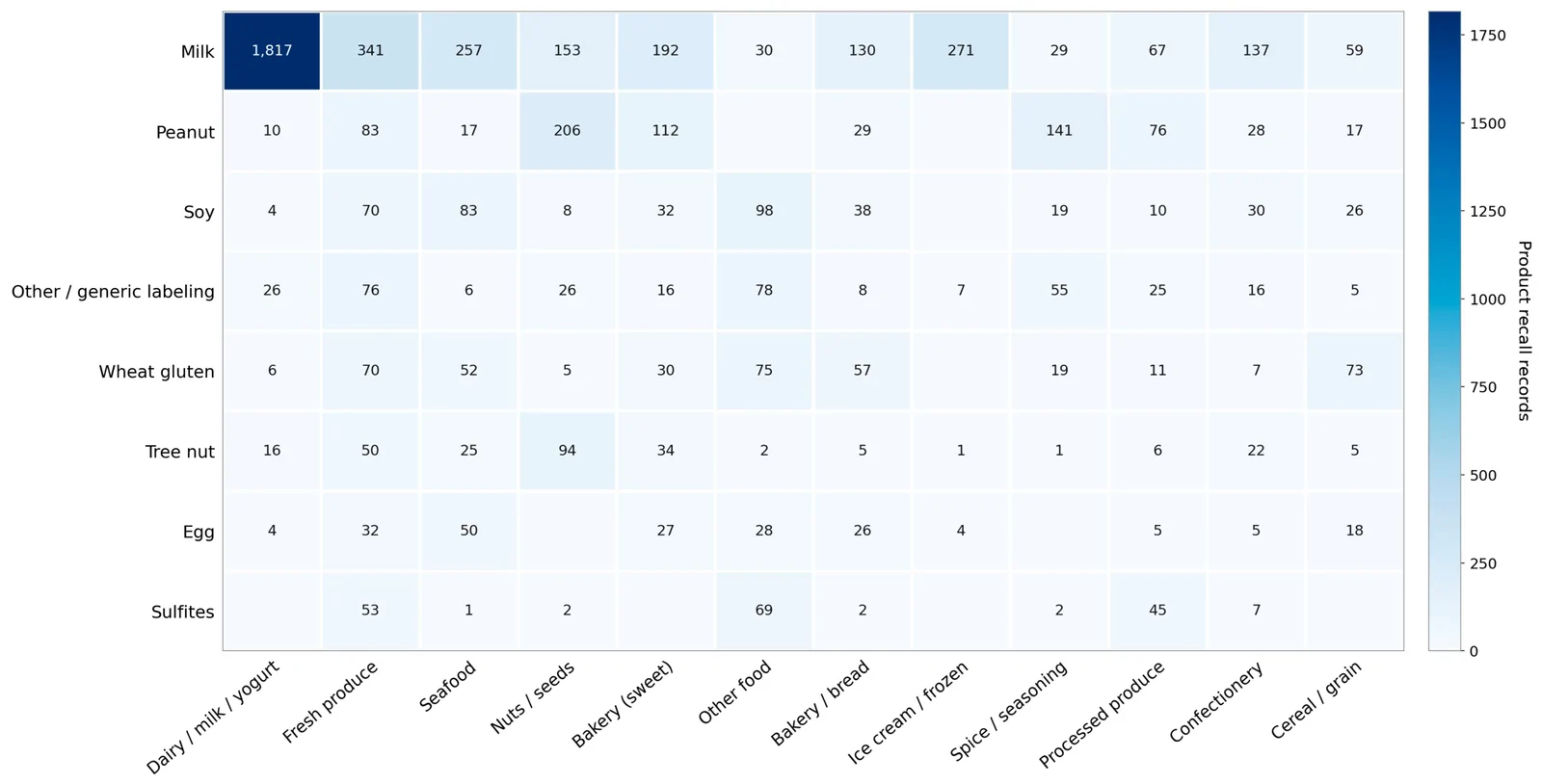
Allergen recalls cross-tabulated by specific allergen and product category, FY2013–2025.

Within the allergen category shown in Figure 6, undeclared milk is overwhelmingly dominant (3688 of 6717 allergen product recall records) and concentrates in dairy/cheese, ice cream, and bakery products, reflecting both milk’s ubiquity as an ingredient and the severity with which undeclared dairy is treated. Peanut and tree-nut recalls cluster in nuts/seeds, snacks, and confectionery. Sesame appears in spice/seasoning and bakery products consistent with its 2023 addition as the ninth major allergen under the FASTER Act [5,6], but its still-modest count (55 records) shows the change has not, within this window, produced an allergen surge large enough to alter the overall hazard ranking.

A multivariable logistic regression on 24,761 classified product recall records (Class I base rate 45.6%), adjusting for hazard category, product group, firm notification method, and distribution reach, identified hazard as the dominant driver: microbiological recalls carried 6.2-fold higher odds of Class I classification than allergen recalls (adjusted odds ratio 6.25, 95% CI 5.82–6.70), while physical hazards were essentially never severe (aOR 0.01) and chemical hazards were markedly less likely than allergens (aOR 0.21).

Recalls announced by press release had 3.4-fold higher odds of being Class I than those communicated only by direct firm contact (aOR 3.38), consistent with firms reserving the most public channel for the most dangerous events; counter-intuitively, nationwide distribution was associated with lower odds (aOR 0.68), because large national recalls are disproportionately undeclared-allergen labeling events whereas the most severe microbiological recalls cluster in regionally distributed fresh and refrigerated foods. A companion gradient-boosting classifier reached a cross-validated ROC-AUC of 0.86, with hazard category by far the most important predictor. Microbiological hazards are therefore not only the most common recalls but, recall for recall, also the most likely to be severe.

### 3.4. Comparison of FDA and USDA-FSIS Food Recalls

**Figure 7:**
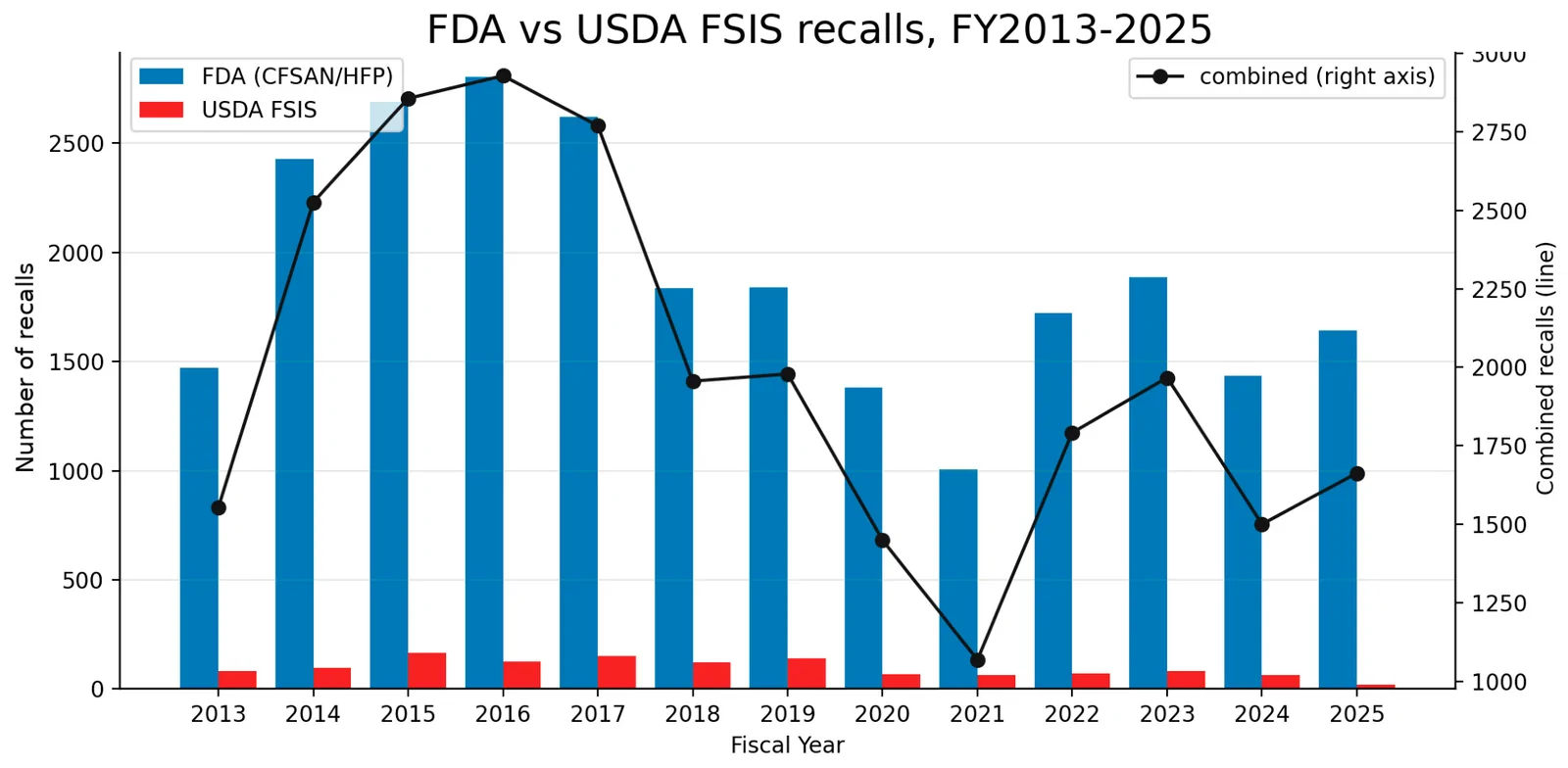
Annual FDA and FSIS recall activity, FY2013–FY2025. FDA bars are product recall records and FSIS bars are recall events, so the two series are plotted at different units of analysis and their heights should not be compared directly. Both agencies show similar year-to-year fluctuation but at different scales (left/right axes), and the combined trend is also non-monotonic.

The FSIS record contained 1248 recall events, compared with 6674 recall events in the FDA record for the same time period—a roughly 5:1 ratio (Figure 7). Compared at the product level instead, those 1248 FSIS events correspond to 24,761 FDA product recall records, a ratio of roughly 20:1; the two ratios describe the same data at different units of analysis and are not interchangeable. This can be partially explained by the fact that the two agencies operate at very different scales: FSIS inspects every animal-slaughter and processing operation continuously (catching most contamination at the plant level before product release), while FDA performs less frequent inspections of a much wider universe of facilities. FSIS recalls are also more severe on average: Class I share is 68.6% at FSIS versus 45.6% at FDA. FSIS additionally uses a fourth designation—Public Health Alert—that has no FDA equivalent. Mann-Kendall trend tests on the FY2013–2025 series gave the same conclusion at both agencies. FDA: tau = −0.33, *p* = 0.127 (no trend); FSIS: tau = −0.46, *p* = 0.033 (decreasing); combined: tau = −0.36, *p* = 0.100 (no trend). The FSIS series should be read with one caveat: the archived FSIS snapshot obtainable at the time of analysis ended 2 March 2025, so FY2025 is a partial year at FSIS while it is complete at FDA, which may steepen the apparent FSIS decline. The ‘food recalls are increasing’ claim does not hold against either agency individually or against the combined record.

It should be noted that the hazard composition at FSIS is structurally different from FDA’s (Table 3). FSIS allergen recalls (38.6%) outnumber microbiological recalls (23.0%)—the opposite of FDA, where microbiological (46.2%) leads allergen (27.1%). The mechanism is plausible: FSIS’s continuous-inspection framework catches microbial issues (*Salmonella*, *E. coli*, *Listeria*) at the plant via HACCP and pathogen-reduction sampling, so most microbial contamination is caught before product enters commerce. Allergen mislabeling, by contrast, is a labeling failure that is not detected at slaughter or processing and almost always shows up at the consumer level—producing a higher allergen-share in the recall record. Physical hazards (bone fragments, foreign material) are also much larger as a share of FSIS recalls (21.7% vs. FDA’s 8.3%) because meat-processing equipment shedding metal/wire/plastic is a major recurring hazard category.

FSIS press-release summaries narrate illness, hospitalization, and death counts inline, but they do so inconsistently. Many summaries omit outcomes altogether because the recall was preventive, and those that report them are not revised as investigations close, so counts parsed from this text understate the true burden by a wide margin and cannot be aggregated across recalls on a common basis. We therefore do not report aggregate FSIS outcome totals. Illness, hospitalization, and death data in this study are drawn from CDC NORS (Section 3.5), which applies a consistent case definition across pathogens and years and is the more reliable source for outcome burden.

### 3.5. Statistical Correlation Between Food Recalls and Foodborne Outbreak Outcomes

**Figure 8:**
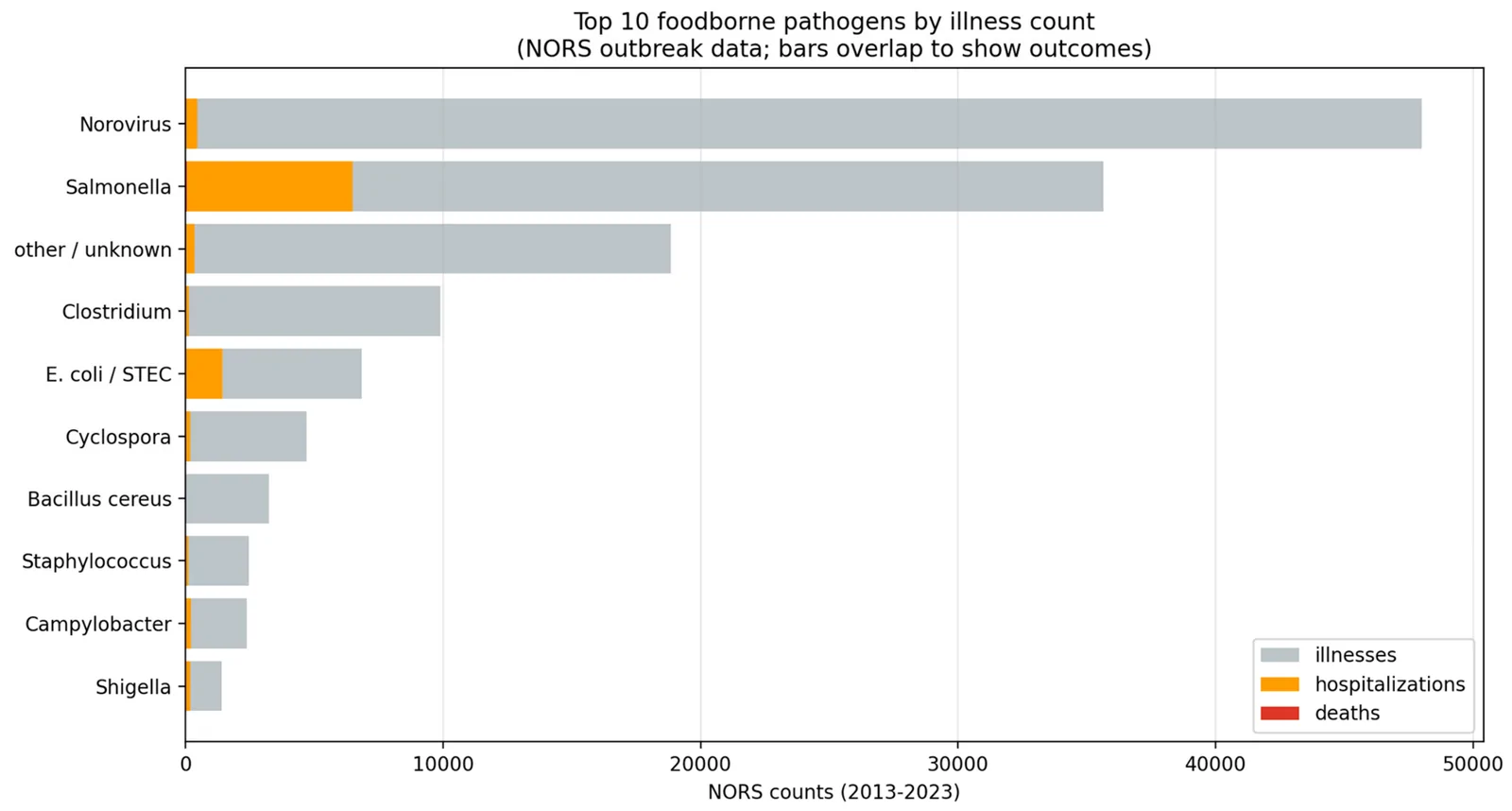
Top 10 foodborne pathogens by illness count, CDC National Outbreak Reporting System (NORS), calendar years 2013–2023. Hospitalization (orange) and death (red) bars are overlaid on illness (gray). The window ends in 2023 because that was the limit of the NORS public-use release at the time of analysis, and is therefore shorter than the FY2013–FY2025 recall window shown in Figure 1, Figure 2, Figure 3, Figure 4, Figure 5, Figure 6 and Figure 7.

We aligned NORS public-use foodborne-outbreak data (data.cdc.gov dataset 5xkq-dg7x) to FDA product recall records for the 2013–2023 window (Figure 8). This window is shorter than the FY2013–FY2025 recall window used in Figure 1, Figure 2, Figure 3, Figure 4, Figure 5, Figure 6 and Figure 7 because the CDC NORS public-use foodborne-outbreak file had been released only through calendar year 2023 at the time of analysis; the recall series could not be truncated to match without discarding two years of otherwise complete FDA and FSIS data. All recall-versus-outbreak comparisons reported in this section, including the Spearman rank correlation, are computed on the shared 2013–2023 period, so no comparison spans mismatched coverage. NORS reports outbreaks, not recalls, so the join is conceptual (at the pathogen × year level) rather than 1:1 [30]. NORS recorded 8205 foodborne outbreaks involving 135,956 illnesses, 10,657 hospitalizations, and 239 deaths. The overall case-fatality rate was 0.18%, and 7.84% of reported illnesses required hospitalization.

The outbreak-surveillance totals expose the gap between regulatory effort and disease burden. Norovirus produces by far the most outbreak illness (47,994 cases) yet is rarely recallable because it spreads chiefly through infected food handlers rather than a contaminated, traceable product lot [35]. *Listeria* sits at the opposite pole: only 837 reported illnesses but 115 deaths, a 13.7% case-fatality rate an order of magnitude above any other common pathogen [31]. *Salmonella* carries the largest hospitalization burden. These divergent profiles are precisely why recall counts, illness counts, and case-fatality rank the same hazards differently.

**Figure 9:**
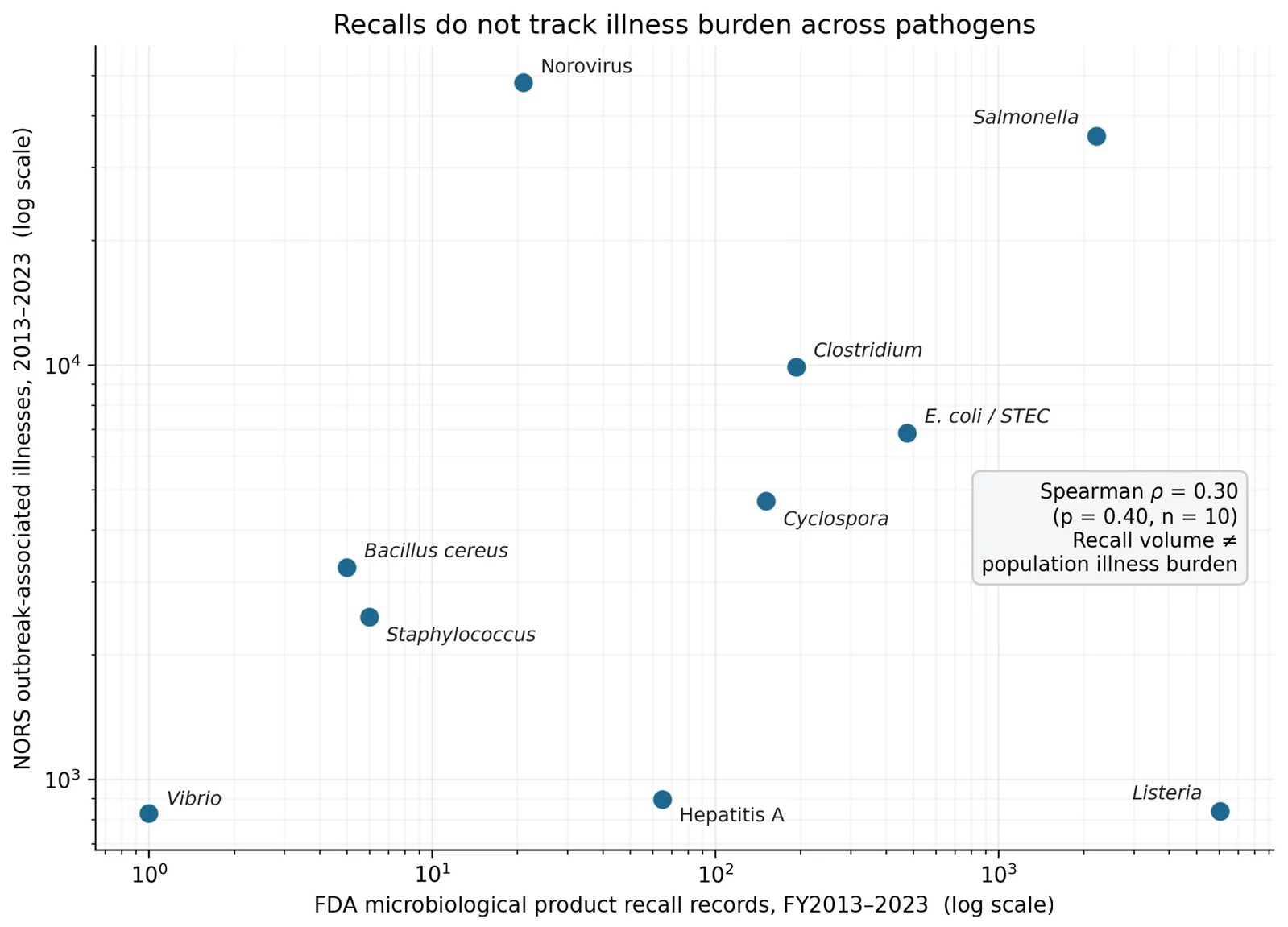
FDA recall counts vs. NORS illnesses per pathogen, both axes log-scaled.

When per-pathogen FDA recall counts are compared to NORS illness, hospitalization, and death counts (Figure 9), the headline finding is a strong decoupling. *Listeria* generated 6056 product recall records over calendar years 2013–2023—the window shared with NORS—but only 837 NORS illnesses, yet *Listeria* caused 115 of the 239 reported foodborne deaths (48%). Norovirus generated only 21 product recall records but 47,994 NORS illnesses, because norovirus typically spreads via infected food handlers in restaurant/catering settings rather than via contaminated manufactured products. *Salmonella* sits between the two—2292 product recall records and 35,638 illnesses, with the largest hospitalization burden of any pathogen (6485). The implication is policy-relevant. Recall counts measure regulatory action, not foodborne disease burden, and the two should not be cited interchangeably [35].

The weak overall rank correlation (Spearman rho = 0.30, not significant) thus reflects a structural feature of how recalls are triggered, not measurement noise: recalls index the regulatory response to severe, product-traceable hazards rather than the population frequency of illness.

With a NORS case-fatality rate of 13.7% in this window (115 deaths in 837 reported illnesses), *Listeria* has the highest case-fatality rate of any common foodborne pathogen, exceeding the next-highest by close to an order of magnitude, and accounted for 48% (115 of 239) of the outbreak-associated foodborne deaths reported to NORS in this window. In absolute national terms, however, non-typhoidal *Salmonella* and *Toxoplasma gondii* are estimated to cause more foodborne deaths each year, so *Listeria*’s distinction is one of lethality per case rather than of total deaths [2,31]. The high recall-to-illness ratio suggests that the FDA’s preventive-recall strategy on *Listeria* is at least partially successful: many product recalls occur before consumer illness can be documented. This is consistent with FDA’s *Listeria*-specific surveillance network and with the post-FSMA emphasis on environmental sampling at refrigerated-food manufacturers. Refrigerated salads, ice cream, and certain soft cheeses are the recurring product categories in both the recall record and the outbreak literature [31].

NORS food-vehicle data identify the foods most often implicated in illness; chicken, onions, cucumbers, ground beef, and turkey lead the list. Note that meat and poultry vehicles dominate the top of the table even though those products are regulated by USDA-FSIS, not FDA; therefore, any ‘all U.S. food safety’ headline requires a parallel FSIS data pull. Among FDA-regulated produce, bagged salad mix, romaine lettuce, cantaloupe, and oysters appear repeatedly [36,37].

Several considerations should be noted about NORS records. Sporadic, isolated, and unreported foodborne illnesses are not captured. CDC estimates that for every reported illness there are many unreported ones, and that *Salmonella* in particular is heavily under-reported [1,2]. A single contamination event can trigger one outbreak record and dozens of product recall records, or vice versa. For this reason, per-pathogen and per-year aggregates, not per-event joins, were used in our analysis. Allergen exposures (anaphylaxis, hospitalizations) are not in NORS at all—NORS is a microbiological/chemical/parasitic-illness system. The illness/death footprint of allergen labeling failures has to come from a different source (e.g., FAACT or Mayo Clinic clinical case series), and this analysis cannot estimate it. USDA-FSIS recalls and outbreaks are excluded from the FDA Food Enforcement record but are present in NORS food-vehicle data.

#### Recall–Outbreak Concordance: Severity, Time, and Geography

To further explore the correlation between food recall and outbreak, we examined three axes: pathogen severity, the timing of recalls relative to outbreaks, and their geography. Recall activity is organized around pathogen severity, hospitalization and death potential, rather than case incidence: the per-pathogen rank correlation between recalls and illnesses is weak, while recalls align with case-fatality and invasive-disease potential. Temporally, recalls follow outbreaks with a lag of roughly two quarters rather than anticipating them, so the system is reactive rather than predictive. Geographically, the apparent alignment between recall and illness counts by state is largely a population artifact: both concentrate in the most populous states, and the correlation nearly vanishes on a per-capita basis.

The recall system therefore behaves as a severity-weighted, reactive instrument, arguably how a recall program should behave, which means recall counts must not be read as a proxy for the public-health burden of foodborne disease. These analyses are necessarily descriptive: NORS captures only the outbreak-associated minority of foodborne illness; preventive recalls that stop an outbreak before it occurs are invisible by construction; and the pathogen cross-section rests on ten data points with wide bootstrap intervals.

Recalls carry a geography in two aspects—where the recalling firm sits (supply side) and where the product was distributed (demand side)—and the openFDA field records the firm’s home state rather than the point of consumer exposure, so this geography maps manufacturing concentration, not consumer risk. California is the dominant origin state (12.4% of records, with a Class I share above 50%), and the high Class I shares in Wisconsin and Maryland track the dairy and refrigerated ready-to-eat footprint. Distribution is broad: roughly a fifth of recalls were nationwide and a further fifth reached international markets, while only about one in seven were confined to a single state.

Combining FDA and FSIS recalls, the leading origin states are California, Illinois, Texas, New York, and Washington, with a secondary Midwest dairy-belt cluster. CDC NORS illness counts by state overlap only partially with recall origin (Ohio and Minnesota rank higher in illness than in firm-state recalls, and Wisconsin the reverse), reinforcing that recall geography and illness geography measure different things, with both concentrating in the most populous states. *Listeria*-related recalls are more geographically focused, clustering in California, Texas, Wisconsin, Washington, and Maryland.

## 4. Discussion

This study provides a comprehensive assessment of US food-recall activity across the post-FSMA period by integrating FDA, USDA-FSIS, and CDC outbreak-surveillance data. Rather than treating recall counts as a direct measure of food-safety deterioration, the findings show that recall activity reflects a combination of hazard severity, regulatory jurisdiction, surveillance capacity, reporting practices, and product traceability. Within this broader context, the results challenge two widely repeated interpretations of recent US food-recall trends. Both popular claims fail when tested directly against the openFDA bulk record. Recall counts were lower in the second half of the window than in the first, a difference supported by the pre/post comparison of annual means (Welch t = 3.08, *p* = 0.024); no individual severity tier shows a significant monotonic trend, with Class II showing the clearest downward tendency. Microbiological hazards remain the largest category by a wide margin. The single year in which allergen exceeded microbiological, FY2017, was driven by a high count of undeclared-milk and undeclared-soy recalls, and the subsequent rapid return to baseline is more consistent with a temporary labeling-related spike than with a sustained shift in the hazard mix [19]. The decreasing classification lag is an FDA-process finding worth flagging separately: it can mimic an ‘increasing recalls’ signal in trackers that count by report_date. These findings do not deny that allergen-related recalls are common or that individual incidents (especially undeclared-milk recalls involving child-targeted products) have outsized public-health weight [38,39]. They do, however, argue for more careful framing in trade press and in regulatory communications: absolute counts and shares should be reported with both numerator and denominator, and time trends should be tested rather than asserted [3].

Several limitations of the FDA dataset should be noted. First, FDA’s unified Human Foods Program began operations on 1 October 2024, corresponding to the start of FY2025. Consequently, the final fiscal year in our analysis crosses an organizational transition in FDA’s human-food oversight [8]. Although the underlying Enforcement Report structure remains comparable, future analyses should assess whether the reorganization affected administrative processing or reporting practices. Second, openFDA does not update a recall’s status after the record has been classified and published; consequently, older entries labeled ‘Ongoing’ cannot be interpreted as current lifecycle indicators [25]. Approximately 7.7% of records remained uncategorized after the 70-rule scheme; however, reassigning the entire uncategorized bucket to allergen would not change the cumulative ranking. Third, product_quantity is free text with mixed units (cases, pounds, bottles, etc.). Volume-based analyses in this report use parsed leading numbers and should be treated as trend indicators only, not unit-consistent volumes. Fourth, the CDC NORS public-use foodborne-outbreak file was available only through calendar year 2023 at the time of analysis, so the recall–outbreak comparisons in Section 3.5 cover 2013–2023 rather than the full FY2013–FY2025 recall window; the two most recent recall years therefore have no matched outbreak-outcome data.

Two recent peer-reviewed studies analyzed US food recalls over long windows, and it is worth asking whether our post-FSMA, supplement-excluded findings agree with them. Our central finding that microbiological hazards, not undeclared allergens, dominate FDA food recalls, independently matches the larger of the two prior studies. Where the studies differ is mostly a matter of how recalls are counted (individual products versus events), which agency is examined (FDA versus USDA), and whether causes are ranked by number of recalls or by pounds of food.

Specifically, our findings align with DeBeer et al. [26], the most directly comparable study, which analyzed more than 35,000 FDA-regulated food and beverage recalls from 2002 to 2023 obtained by FOIA. That study likewise concluded that microbiological contamination, not undeclared allergens, was the leading cause of FDA recalls, with *L. monocytogenes* the single largest individual contributor (their 22% of all recalls). Undeclared allergens accounted for roughly 28% as a whole category, so the comparison supporting microbiological dominance is category against category, not *Listeria* against allergens. Our independent analysis, using a different extraction, analytical window, and hazard-classification method (a transparent, QC-validated hazard classifier; the post-FSMA-core FY2013–FY2025 window with dietary supplements and cosmetics removed), reaches the same ranking: microbiological hazards are the largest category in 12 of 13 years and lead allergen recalls cumulatively by roughly 1.7-fold. That two analyses with different data extractions, windows, and classification schemes converge on the same conclusion materially strengthens the case that the popular ‘allergens are the number-one recall cause’ framing is an artifact of single-year snapshots rather than a durable feature of the record. Both studies also agree that recalls rose around FSMA implementation (2011) and did not continue rising monotonically thereafter, and that Class I (most severe) recalls make up close to half of the total (our Class I base rate 45.6%) [26].

The apparent disagreements among recall studies dissolve along three axes. First, *scale*: DeBeer et al. count individual recalled products (more than 35,000 from roughly 10,000 events, about 3.5 products per event) and we likewise count product recall records (24,761 from 6674 events, 3.71 per event), so the two studies share a counting unit and the difference in absolute totals reflects their longer 2002–2023 span rather than a different basis; the cause *ranking* is unaffected. Second, *scope and window*: Our removal of dietary supplements and cosmetics, together with the later analytical window, may partly explain the lower allergen share and wider microbiological lead. Third, *incidents versus pounds*: the companion USDA study, Blickem et al. [27], shows that at FSIS undeclared allergens are the most common cause by incident count (~33%) while microbiological contamination dominates by *weight* (~59%, led by *Listeria* with Shiga-toxin-producing *E. coli* second), so whether allergens or pathogens are ‘number one’ can flip with the denominator. Our FDA analysis is consistent with the Blickem FDA-versus-FSIS contrast: FSIS skews far more heavily to Class I (their 76%) and to a beef-driven *E. coli* profile, whereas the FDA record is *Salmonella*-heavier and spans a much wider product range [27].

Neither of the two most directly comparable studies extended its analysis beyond descriptive tabulation. To our knowledge, the following applications have not previously been reported for the US food-recall record: (1) A multivariable *severity model* quantifies that microbiological hazards carry roughly six-fold higher adjusted odds of Class I classification than allergens, moving the field’s observation that pathogens are more severe from a stated fact to an adjusted, effect-sized estimate (model ROC-AUC 0.86). (2) A *responsiveness model* shows FDA classifies recalls in a median of about five weeks and has been accelerating by roughly two days per year, with mandated recalls taking markedly longer, an agency-process dimension absent from prior recall papers. (3) A *firm-concentration* analysis quantifies, via a Gini coefficient of 0.70 and a top-decile share of 63%, that recall burden is highly concentrated in a small set of repeat firms whose recall events are also more severe and more microbiological. (4) A *seasonality* analysis formally tests within-year cycles by hazard, showing microbiological recalls are the most evenly distributed across the year. Together with our linkage of recalls to CDC NORS outbreak outcomes and our recall-duration survival analysis, these extensions reframe the recall record from a descriptive tally into an analyzed system of severity, timing, responsiveness, and accountability.

As FSMA represented the most extensive reform of US food-safety law in several decades [40], we asked whether it left a visible mark on recalls, but a clean causal test is not identified. The openFDA record is densely populated only from 2012 onward, after FSMA’s 2011 enactment, so there is no usable pre-law baseline, and a difference-in-differences design using USDA-FSIS as an untreated control fails because the two systems did not move in parallel beforehand (interaction *p* = 0.16). We therefore traced FSMA’s two most direct levers on recalls.

First, Section 206 gave FDA authority to order a food recall after a responsible party declined an opportunity to conduct an adequate voluntary recall [41]. In our dataset, this authority was associated with only 7 of 6674 recall events (0.10%), supporting its characterization as a rarely used backstop rather than a major contributor to recall counts. Second, the microbiological share of recalls did fall (from 51.6% in calendar 2012–2015 to 44.3% in calendar 2017–2025, *p* < 0.001; this mechanism-tracer comparison is computed on calendar years across the full cleaned export rather than on the FY2013–FY2025 analysis window, so as to reach back to the pre-rule period). This direction is consistent with the intended effect of the Preventive Controls for Human Food rule, which requires facilities to identify and implement controls for known or reasonably foreseeable hazards [42,43]. However, the change is non-monotonic and confounded: it coincides with a contemporaneous surge in undeclared-allergen recalls (peaking near 50% in 2017) and with the progressive adoption of whole-genome sequencing in PulseNet, beginning with *Listeria* investigations in 2013 and expanding to routine surveillance of additional foodborne pathogens during 2015–2019. The adoption of WGS improved the detection and linkage of outbreak-associated cases and food isolates, potentially increasing the identification of microbiological events and thus acting in the opposite direction from preventive controls [44,45]. With a preventive force and a detection force acting in opposite directions over the same years, the record cannot isolate either. This is not evidence that FSMA failed, as prevention that stops contamination before it occurs would never appear in recall data, but it cautions against reading recall trends as a scorecard for the Act.

## 5. Conclusions

Across three independent data sources: the openFDA Food Enforcement export, the USDA-FSIS recall record, and CDC NORS outbreak surveillance covering FY2013–FY2025, neither popular claim survives testing. Total US food recalls did not rise across the post-FSMA-core period: counts peaked in FY2015–FY2016 and ran materially lower thereafter, a decline supported by the pre/post comparison of annual means (Welch t = 3.08, *p* = 0.024) rather than by a monotonic trend in any single severity tier. Microbiological hazards, not undeclared allergens, are the dominant cause of FDA food recalls. They were the largest category in 12 of 13 years and roughly 1.7 times more common than allergens cumulatively, and each microbiological recall was also far more likely to be classified as severe. Allergen labeling failures are an important hazard and lead recall counts at FSIS, but at FDA they peaked once (FY2017) and regressed rather than displacing pathogens. *L. monocytogenes* is the lethal outlier: relatively few documented illnesses but the highest case-fatality of any common foodborne pathogen and the single largest driver of FDA microbiological recalls, a pattern consistent with a preventive-recall posture that removes contaminated product before illness in most years.

These findings carry a single methodological lesson. Recall counts, outbreak illness counts, and case-fatality measure different things and rank the same hazards differently, sometimes oppositely, so the three should not be cited interchangeably. The shortening classification lag, the censoring artifacts in recall-duration data, the replication of event-level totals across product recall records, and the population scaling of state-level geography each show how a raw count or average, read without regard to how the data are generated, tells a more dramatic story than the data support. Public-health framing of food recalls should test trends against the bulk record rather than infer them from periodic alert volume and should report both numerator and denominator. Read with these corrections, the recall record describes a maturing, severity-weighted surveillance system, not a deteriorating food supply.

## Figures and Tables

**Table 1:** Dataset characteristics.

Field	Value
Data source	openFDA Food Enforcement bulk export
Snapshot date	9 May 2026
Records, full export	28,784
Cosmetic recalls removed	5
Animal/pet-food recalls removed	2
Dietary-supplement recalls removed	1804
Conventional human-food records	26,973
Date anchor	recall_initiation_date
Analysis window (fiscal year)	FY2013–FY2025
Product recall records in analysis window	24,761
Unique recalling firms	4649
Median classification lag (days)	38
90th-percentile classification lag (days)	126

**Table 2:** Recall events, product recall records, and products per event by fiscal year, FY2013–FY2025.

Fiscal Year	Recall Events	Product Recall Records	Products per Event
FY2013	514	1473	2.87
FY2014	507	2429	4.79
FY2015	589	2691	4.57
FY2016	714	2802	3.92
FY2017	623	2620	4.21
FY2018	533	1835	3.44
FY2019	503	1839	3.66
FY2020	424	1381	3.26
FY2021	403	1005	2.49
FY2022	455	1722	3.79
FY2023	445	1886	4.24
FY2024	435	1434	3.30
FY2025	529	1643	3.11
**All years**	**6674**	**24,761**	**3.71**

Note. Fiscal-year product-record counts sum to 24,760. One record in the analysis window carries an unusable recall_initiation_date and therefore cannot be assigned to a fiscal year; it is included in the 24,761 total reported in Table 1 and Table 3 and excluded from the classification-lag analysis (*n* = 24,760).

**Table 3:** Hazard category mix: FDA vs. FSIS.

Hazard Category	FDA (n)	FDA (%)	FSIS (n)	FSIS (%)
Microbiological	11,443	46.2	287	23.0
Allergen	6717	27.1	482	38.6
Chemical	1023	4.1	10	0.8
Physical	2062	8.3	271	21.7
Other/quality	1617	6.5	52	4.2
Uncategorized	1899	7.7	146	11.7
**Total**	**24,761**	**100.0**	**1248**	**100.0**

Note. Values are counts (n) and column percentages for the FY2013–FY2025 window. FDA counts are product recall records and FSIS counts are recall events, so the two columns describe the same period at different units of analysis and their absolute totals are not directly comparable. Percentages may not sum to 100 owing to rounding. FDA = Food and Drug Administration; FSIS = USDA Food Safety and Inspection Service. FSIS Public Health Alerts, which have no FDA equivalent, are not counted as recalls and are excluded.

## Data Availability

All data analyzed in this study are made publicly available by U.S. FDA, CDC, and USDA FSIS.
